# Comparing Early and Long-Term Outcomes of “Truly Autonomous” Senior Resident-Led with Consultant-Led Cardiac Surgery: A 10-Year Propensity-Matched Study

**DOI:** 10.1093/icvts/ivag099

**Published:** 2026-04-09

**Authors:** Ujjawal Aditya Kumar, Eteesha Rao, Fadi Ibrahim Al-Zubaidi, Aravinda Page, Harry Smith, Daniel Sitaranjan, Ravi De Silva, Shakil Farid

**Affiliations:** Department of Cardiac Surgery, Royal Papworth Hospital, Cambridge CB2 0AY, United Kingdom; School of Clinical Medicine, University of Cambridge, Cambridge CB2 0SP, United Kingdom; Department of Cardiac Surgery, Royal Papworth Hospital, Cambridge CB2 0AY, United Kingdom; Medical School, Newcastle University, Newcastle-upon-Tyne NE2 4HH, United Kingdom; Department of Cardiac Surgery, Royal Papworth Hospital, Cambridge CB2 0AY, United Kingdom; Medical Sciences Division, University of Oxford, Oxford OX3 9DU, United Kingdom; Department of Cardiac Surgery, Oxford University Hospitals, Oxford OX3 9DU, United Kingdom; Department of Cardiac Surgery, Royal Papworth Hospital, Cambridge CB2 0AY, United Kingdom; Department of Cardiac Surgery, Harefield Hospital, Uxbridge UB9 6JH, United Kingdom; Department of Cardiac Surgery, Royal Papworth Hospital, Cambridge CB2 0AY, United Kingdom; Department of Cardiac Surgery, Hammersmith Hospital, London W12 0HS, United Kingdom; Department of Cardiac Surgery, Royal Papworth Hospital, Cambridge CB2 0AY, United Kingdom; Department of Cardiac Surgery, Morriston Hospital, Swansea SA6 6NL, United Kingdom; Department of Cardiac Surgery, Royal Papworth Hospital, Cambridge CB2 0AY, United Kingdom; Department of Cardiac Surgery, Royal Papworth Hospital, Cambridge CB2 0AY, United Kingdom

**Keywords:** surgical training, outcomes, mortality, morbidity

## Abstract

**Objectives:**

Cardiac surgery demands substantial technical skill and intraoperative judgement. Residents must develop operative autonomy in preparation for independent consultant practice. However, current challenges, including working hour restrictions, shorter training programmes, reduced operative exposure, and increasing case complexity, limit opportunities for skill development. This study evaluated the safety of “truly autonomous” cardiac surgery performed by senior residents without direct consultant supervision.

**Methods:**

Data for all adult cardiac surgeries between January 2015 and December 2024 were extracted from our institutional database. All resident-led cases undertaken without direct consultant supervision (group R) were identified and 1:1 propensity-score matched with consultant-led cases (group C) using the European System for Cardiac Operative Risk Evaluation (EuroSCORE) II covariates. In-hospital outcomes (mortality, complications, length of stay) and long-term survival were compared.

**Results:**

A total of 16 945 procedures were undertaken during the study period. After applying inclusion and exclusion criteria, matching yielded 803 pairs, giving a study population of 1 606 patients. Groups had comparable demographics, preoperative characteristics, risk scores and cardiopulmonary bypass and cross-clamp times. Consultants undertook significantly more combined and aortic cases, with residents performing more isolated CABG and isolated valve procedures. Groups had similar in-hospital outcomes and long-term survival. In a subgroup analysis of emergency operations, groups had similar outcomes.

**Conclusions:**

Truly autonomous cardiac surgery by senior residents demonstrated comparable in-hospital and post-discharge outcomes to consultant-led cases. Even in emergency procedures, senior residents achieved comparable outcomes to matched consultant-led cases. Our study shows truly autonomous operating in appropriately selected cases to be feasible and safe, providing evidence-based justification for progressive independence in cardiac surgical training.

## Graphical abstract

**Figure:**
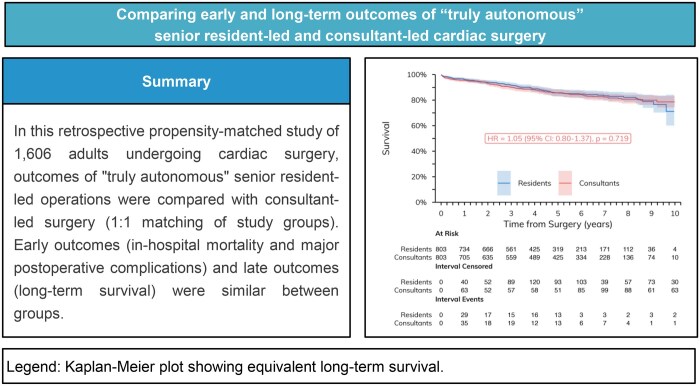

## INTRODUCTION

Cardiac surgical training has undergone significant transformations globally.^1^ UK programmes can now recruit residents 2 years after medical school into fully integrated pathways without prior surgical training, with the 2021 curriculum further reducing training duration from 8 to 7 years. These changes limit residents’ broad surgical exposure and operative volumes, which is particularly challenging in cardiac surgery given its increasing procedural complexity and patient comorbidities.^2^ Residents now spend less consecutive time with mentors, eroding the trust-based longitudinal model that traditionally enabled autonomous surgery in later training years.^3^ Compounding these issues, worldwide restrictions on working hours, including European Working Time Directive-driven shift patterns, have curtailed operative exposure.^4^^,^^5^

“Skin-to-skin” operative experience remains essential for day 1 consultant readiness,^6^ although decreasing surgical volumes constrains opportunities. Annual case numbers have decreased due to factors such as increases in transcatheter interventions and outcome scrutiny.^7^ Despite improvements in recent years, national volume still trails pre-pandemic levels. Mitigation requires careful case selection, prioritizing curriculum-defined index procedures for progressive, supervised involvement.

The transition to independent practice is pivotal in cardiothoracic training, challenged by declining autonomy, patient safety concerns, and variable faculty entrustment.^8^ To facilitate this transition, senior residents require experience in autonomous operating, though this must not compromise patient safety. Evidence from other surgical specialities suggests that senior residents achieve comparable outcomes to consultants in appropriately selected core procedures.^9^ Structured autonomy, entrustment, and competency assessments are key, particularly in the modern era of cardiac surgery.

Prior comparisons remain limited by short follow-up,^10–12^ small samples,^13^ selection bias,^10^ and direct supervision. We have previously shown that residents can safely perform cardiac surgery with direct consultant supervision, both in curriculum-aligned index cases^14^ and in the emergency setting.^15^

This study sought to address this by evaluating the safety and long-term efficacy of “truly autonomous” cardiac surgical procedures performed by senior residents at a high-volume quaternary centre over 10 years.

## PATIENTS AND METHODS

### Study population

All adult patients undergoing cardiac surgery at our institution between 2015 and 2024 were included. Complex cases undertaken with institutional collective responsibility were excluded. Patients undergoing “salvage” surgery (requiring resuscitation prior to anaesthesia induction) were excluded. Miscellaneous cardiac cases that were not categorised into coronary artery bypass grafting, valve surgery, major aortic surgery, or a combination of the above were also excluded. The study cohort was stratified based on whether a surgical resident (group R) or a consultant (group C) performed the critical operative steps, consistent with national operative responsibility criteria. Our institutional policy is that for truly autonomous resident-led cases, either a more junior resident or a surgical care practitioner/surgeon’s assistant acts as the first assistant, with the supervising consultant not scrubbed if all is progressing well. If the consultant does scrub in at any point, either due to intraoperative complications or in order to facilitate operative progress, this does not count as a truly independent, autonomous case. An emergency surgery subgroup (unscheduled patients with ongoing refractory cardiac compromise) was defined, matched, and analysed.

### Data sources

Data were retrieved from our institutional registry for all included patients. Survival data were obtained from the UK National Patient Administration System on March 1, 2025.

### Ethical approval

All patients gave informed written consent for surgical procedures and resident involvement. The Royal Papworth Hospital Research and Development Department waived formal ethical approval requirements (Non-HRA0081, STA-381) and the requirement for specific patient consent, as only anonymised data from routine patient care were used. Patients who declined health record sharing through the NHS National Data Opt-Out were excluded from our initial patient sample. This study was reported in accordance with the STROBE guidelines.^16^

### Surgical training

Our centre has a long-standing residency programme that recruited residents with prior general surgical experience until 2015. Since then, only integrated residents have been appointed, undertaking rotations in related specialities (eg, general, vascular, plastic surgery, critical care). Training is supplemented by a local curriculum of wet lab, dry lab and didactic teaching as well as national courses incorporating high-fidelity simulation.^17^

Resident competency is systematically assessed through workplace-based assessments across multiple clinical settings. Consultant-level supervisors evaluate theoretical knowledge and operative skills. Only in the final months of residency are residents typically allowed to perform independent cases, once consultants are familiar with their capabilities as being safe to operate without direct supervision.

### Follow-up and outcomes

In-hospital outcomes were all-cause in-hospital mortality, rates of return-to-theatre, deep sternal wound infection, neurological events (stroke/transient ischaemic attack), renal failure requiring renal replacement therapy, and the duration of postoperative hospitalization.

Post-discharge outcomes were mortality (1 and 6 months, 1, 3, and 5 years) and long-term survival (Kaplan-Meier method).

### Statistical analysis

Analyses were performed using R v4.5.0, with propensity-score matching and Kaplan-Meier analyses performed using “MatchIt” and “survival,” respectively. Propensity matching was conducted as previously described by our group.^14^^,^^15^ Poor mobility and pulmonary hypertension were excluded from the matching model due to incomplete data (>5%). There were no other missing data, as these were handled by listwise deletion prior to dataset provision.

Normally distributed data are presented as mean and standard deviation, and the *t*-test is used for comparing groups. Other continuous data are presented as a median and interquartile range, with the Mann-Whitney *U*-test used to compare groups. Categorical variables are presented as *N* (%), and groups were compared using either the chi-squared or the Fisher exact test if the expected frequency was ≤5. *P*-values were 2-tailed at the .05 level without multiplicity adjustment.

## RESULTS

**Figure 1.**
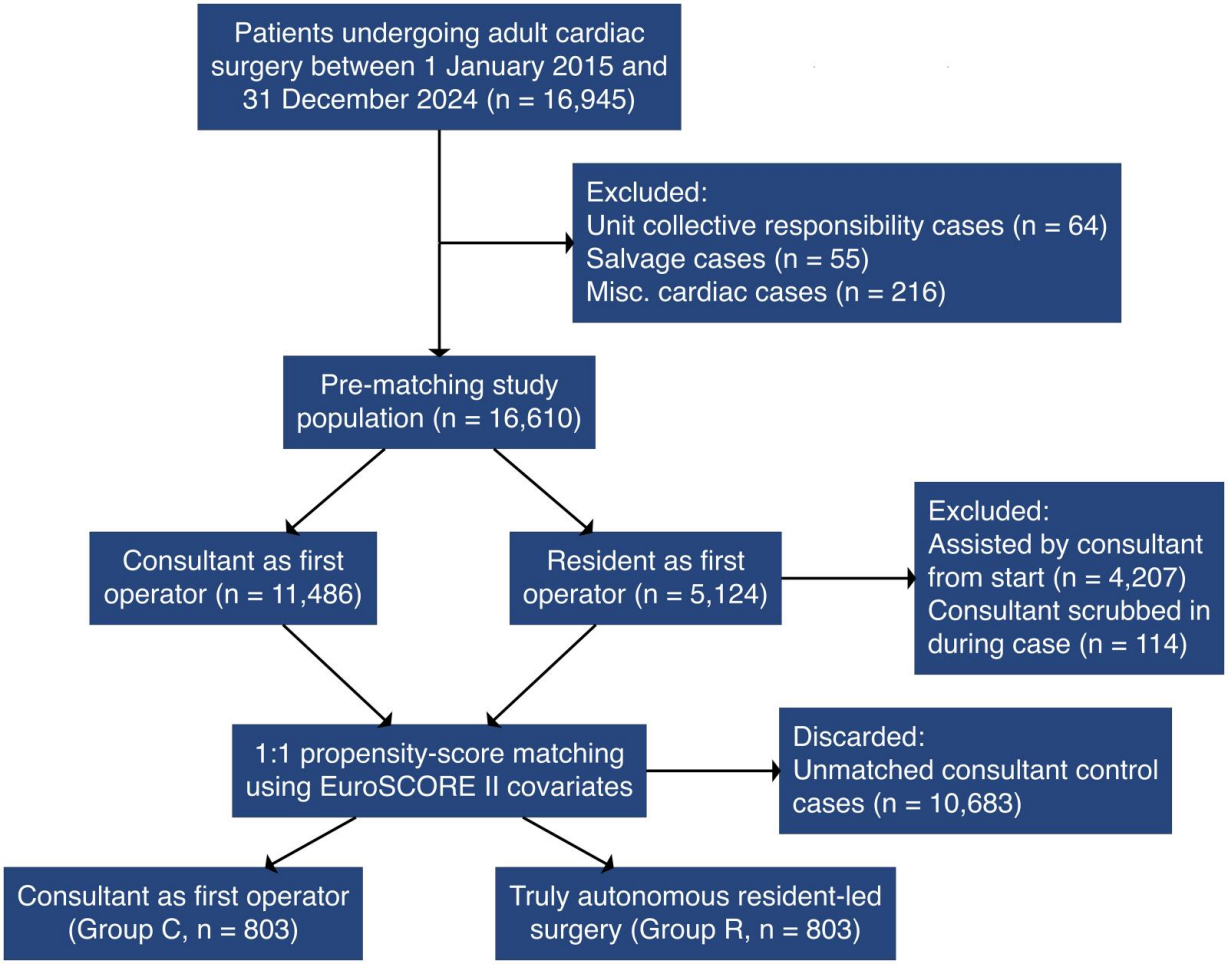
Patient Selection Flowchart

Between 2015 and 2024, 16 945 patients underwent adult cardiac surgery at our institution. After applying exclusion criteria, 16 610 patients were included (**Figure 1**), and resident-led procedures (*n* = 5 124) were identified. Most of these cases involved a consultant scrubbed in and assisting the resident from the start of the operation (*n* = 4 207). In a further 114 cases, a consultant scrubbed in partway through the case to assist due to intraoperative complications or to facilitate operative progress. Excluding all cases involving consultant scrubbed participation left 803 procedures in which the resident completed the operation without a consultant scrubbed at any stage, constituting the “truly autonomous” group. After propensity matching these with consultant-led controls, the final study cohort comprised 1 606 patients.

### Preoperative characteristics

Before propensity matching, there were several significant differences in preoperative characteristics between resident-led and consultant-led cases (**Table 1**). Residents operated on older patients (69 vs 68 years, *P* = .014), fewer females (22.5% vs 29.9%, *P* < .001), and patients with significantly lower surgical risk as reflected by European System for Cardiac Operative Risk Evaluation (EuroSCORE) II (median 1.73 vs 2.17, *P* < .001). Residents performed fewer reoperations (previous cardiac surgery: 0.7% vs 7.6%, *P* < .001), multivalve procedures (1.7% vs 6.9%), or isolated valve/aortic surgery and more frequently performed isolated coronary artery bypass grafting (CABG) (56.5% vs 32.3%, all *P* < .001).

**Table 1.** Preoperative Characteristics

Variable	Unmatched data				Matched data			
	Residents *N* = 803	Consultants *N* = 11 486	*P*-value	SMD	Group R (residents) *N* = 803	Group C (consultants) *N* = 803	*P*-value	SMD
Age (years)	69 ± 9	68 ± 12	.014	0.080	69 ± 9	69 ± 10	.854	0.009
Sex: female	181 (22.5%)	3431 (29.9%)	<.001	0.167	181 (22.5%)	171 (21.3%)	.587	0.030
Chronic lung disease	137 (17.1%)	1862 (16.2%)	.561	0.023	137 (17.1%)	122 (15.2%)	.342	0.051
Extracardiac arteriopathy	75 (9.3%)	1108 (9.6%)	.824	0.010	75 (9.3%)	71 (8.8%)	.795	0.017
Previous cardiac surgery	6 (0.7%)	870 (7.6%)	<.001	0.347	6 (0.7%)	5 (0.6%)	>.999	0.015
1	6 (0.7%)	715 (6.2%)			6 (0.7%)	5 (0.6%)		
2	0 (0.0%)	123 (1.1%)			0 (0.0%)	0 (0.0%)		
3	0 (0.0%)	9 (0.1%)			0 (0.0%)	0 (0.0%)		
4	0 (0.0%)	16 (0.1%)			0 (0.0%)	0 (0.0%)		
5	0 (0.0%)	2 (0.0%)			0 (0.0%)	0 (0.0%)		
6	0 (0.0%)	5 (0.0%)			0 (0.0%)	0 (0.0%)		
Critical preoperative state	8 (1.0%)	477 (4.2%)	<.001	0.200	8 (1.0%)	9 (1.1%)	>.999	0.012
Preoperative CrCl (mL/minute)	83 (63-106)	79 (59-102)	.001	0.097	83 (63-106)	83 (62-103)	.706	0.039
Renal impairment	396 (49.3%)	6237 (54.3%)	.015	0.116	396 (49.3%)	389 (48.4%)	.258	0.049
CrCl 50-85 mL/minute	307 (38.2%)	4589 (40.0%)			307 (38.2%)	317 (39.5%)		
CrCl <50 mL/minute	80 (10.0%)	1452 (12.6%)			80 (10.0%)	69 (8.6%)		
Dialysis	9 (1.1%)	196 (1.7%)			9 (1.1%)	3 (0.4%)		
Diabetes mellitus	229 (28.5%)	2819 (24.5%)	.084	0.089	229 (28.5%)	216 (26.9%)	.512	0.057
Lifestyle controlled	43 (5.4%)	568 (4.9%)			43 (5.4%)	48 (6.0%)		
Oral medication	125 (15.6%)	1508 (13.1%)			125 (15.6%)	121 (15.1%)		
Insulin dependent	61 (7.6%)	743 (6.5%)			61 (7.6%)	47 (5.9%)		
CCS class			<.001	0.239			.353	0.088
1	347 (43.2%)	6546 (57.0%)			347 (43.2%)	378 (47.1%)		
2	223 (27.8%)	2408 (21.0%)			223 (27.8%)	221 (27.5%)		
3	171 (21.3%)	1882 (16.4%)			171 (21.3%)	150 (18.7%)		
4	62 (7.7%)	650 (5.7%)			62 (7.7%)	54 (6.7%)		
LV dysfunction	225 (28.0%)	3846 (33.5%)	.001	0.152	225 (28.0%)	215 (26.8%)	.851	0.039
Mild dysfunction	192 (23.9%)	3016 (26.3%)			192 (23.9%)	188 (23.4%)		
Moderate dysfunction	26 (3.2%)	604 (5.3%)			26 (3.2%)	22 (2.7%)		
Severe dysfunction	7 (0.9%)	226 (2.0%)			7 (0.9%)	5 (0.6%)		
Previous myocardial infarction	308 (38.4%)	2972 (25.9%)	<.001	0.270	308 (38.4%)	276 (34.4%)	.108	0.083
Myocardial infarction within 90 d	217 (27.0%)	1989 (17.3%)	<.001	0.235	217 (27.0%)	208 (25.9%)	.651	0.025
NYHA class			.172	0.078			.968	0.009
1	192 (23.9%)	2535 (22.1%)			192 (23.9%)	185 (23.0%)		
2	333 (41.5%)	4600 (40.0%)			333 (41.5%)	339 (42.2%)		
3	240 (29.9%)	3636 (31.7%)			240 (29.9%)	243 (30.3%)		
4	38 (4.7%)	715 (6.2%)			38 (4.7%)	36 (4.5%)		
Body mass index (kg/m^2^)	30.80 ± 23.41	29.34 ± 17.61	.087	0.070	30.80 ± 23.41	29.77 ± 19.13	.336	0.048
Smoking history	473 (58.9%)	5959 (51.9%)	<.001	0.146	473 (58.9%)	457 (56.9%)	.670	0.045
Ex-smoker	392 (48.8%)	5064 (44.1%)			392 (48.8%)	383 (47.7%)		
Current smoker	81 (10.1%)	895 (7.8%)			81 (10.1%)	74 (9.2%)		
Neurological dysfunction	76 (9.5%)	1050 (9.1%)	.808	0.011	76 (9.5%)	59 (7.3%)	.150	0.076
Hypertension	482 (60.0%)	6779 (59.0%)	.601	0.020	482 (60.0%)	481 (59.9%)	>.999	0.003
Unstable angina	32 (4.0%)	393 (3.4%)	.472	0.029	32 (4.0%)	25 (3.1%)	.418	0.047

Abbreviations: CCS, Canadian Cardiovascular Society; CrCl, creatinine clearance; LV, left ventricle; NYHA, New York Heart Association; SMD, standardised mean difference.

Residents also operated on patients with lower rates of critical preoperative state (1.0% vs 4.2%, *P* < .001), left ventricular dysfunction (28.0% vs 33.5%, *P* = .001), and renal impairment, but higher rates of recent (≤90 days) myocardial infarction (38.4% vs 25.9%, *P* < .001) and any previous myocardial infarction (27.0% vs 17.3%, *P* < .001). Cardiopulmonary bypass and aortic cross-clamp times were shorter in resident-led cases (95 vs 99 min, *P* = .027; 63 vs 66 min, *P* = .004).

However, after matching, groups were well-balanced (**Table 1** and **Supplementary Figure 1**). All previously significant differences were eliminated, with no significant differences in demographics, comorbidities, functional status or operative risk score (**Table 2**).

**Table 2.** Operative Characteristics

Variable	Unmatched data			Matched data		
	Residents *N* = 803	Consultants *N* = 11 486	*P*-value	Group R (residents) *N* = 803	Group C (consultants) *N* = 803	*P*-value
EuroSCORE II	1.73 (1.07-2.95)	2.17 (1.18-4.53)	<.001	1.73 (1.07-2.95)	1.67 (1.07-2.94)	.960
Urgency			<.001			.012
Elective	444 (55.3%)	7268 (63.3%)		444 (55.3%)	484 (60.3%)	
Urgent	339 (42.2%)	3384 (29.5%)		339 (42.2%)	287 (35.7%)	
Emergency	20 (2.5%)	834 (7.3%)		20 (2.5%)	32 (4.0%)	
Procedure type			<.001			<.001
Aortic	38 (4.7%)	1387 (12.1%)		38 (4.7%)	36 (4.5%)	
CABG	454 (56.5%)	3712 (32.3%)		454 (56.5%)	372 (46.3%)	
CABG + valve	117 (14.6%)	1902 (16.6%)		117 (14.6%)	177 (22.0%)	
Valve	180 (22.4%)	3691 (32.1%)		180 (22.4%)	178 (22.2%)	
Multivalve	14 (1.7%)	794 (6.9%)		14 (1.7%)	40 (5.0%)	
Median number of distal anastomoses	3 (2-3)	3 (2-3)	.546	3 (2-3)	3 (2-3)	.381
Cardiopulmonary bypass time (min)	95 (77-120)	99 (74-134)	.027	95 (77-120)	96 (74-124)	.972
Aortic cross-clamp time (min)	63 (49-82)	66 (48-91)	.004	63 (49-82)	63 (47-83)	.928

Abbreviations: CABG, coronary artery bypass graft; EuroSCORE, European System for Cardiac Operative Risk Evaluation.

### Operative characteristics

Operative characteristics of the study groups are given in **Table 2**. As expected, operative risk scores were similar, as was the weight of operation defined for EuroSCORE II (*P* = .846). Consultants performed more combined procedures (CABG + Valve and Multivalve), whereas residents undertook more isolated CABG cases. Cardiopulmonary bypass (CPB) and aortic cross clamp (ACC) times were similar between groups R and C, with comparable numbers of distal anastomoses. Residents performed more in-house urgent cases, whereas consultants performed more elective and emergency procedures.

### Outcomes

**Figure 2:**
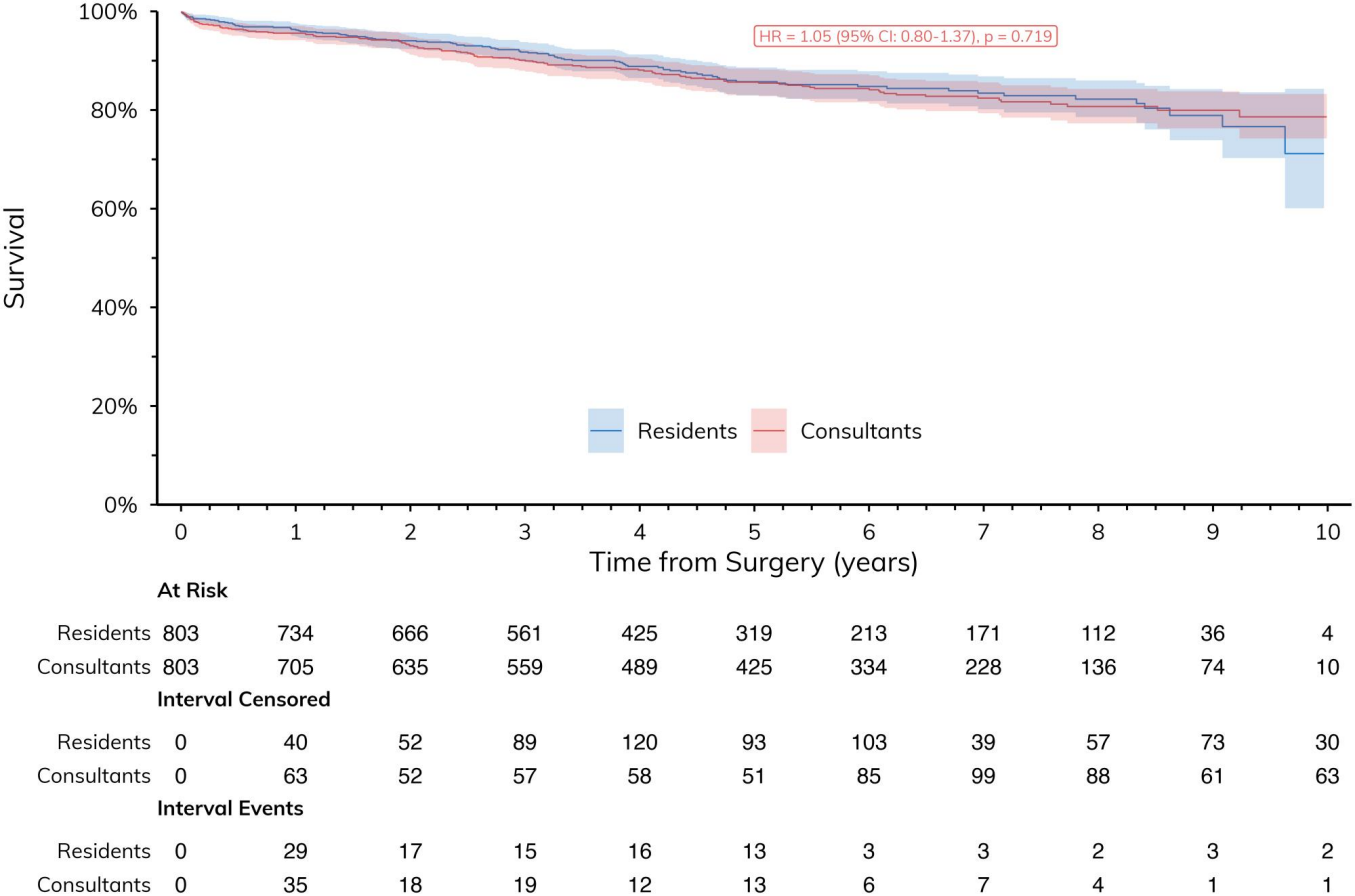
Comparison of Long-Term Survival. Median follow-up duration was 54.6 months (IQR 30.8-83.9)

Outcomes are shown in **Table 3**. In-hospital outcomes (mortality, duration of postoperative hospitalization and complication rates) were similar between resident and consultant-led procedures. Long-term survival (**Figure 2**) up to 10 years was similar between the 2 groups (HR = 1.05, 95% confidence interval [CI]: 0.80-1.37, *P* = .719).

**Table 3.** Outcomes

Variable	Unmatched data			Matched data		
	Residents *N* = 803	Consultants *N* = 11 486	*P*-value	Group R (residents) *N* = 803	Group C (consultants) *N* = 803	*P*-value
In-hospital outcomes						
Hospital mortality	6 (0.7%)	351 (3.1%)	<.001	6 (0.7%)	12 (1.5%)	.236
Return to theatre	18 (2.2%)	415 (3.6%)	.657	18 (2.2%)	19 (2.4%)	.416
Deep sternal wound infection	11 (1.4%)	89 (0.8%)	.107	11 (1.4%)	7 (0.9%)	.477
Stroke	7 (0.9%)	191 (1.7%)	.115	7 (0.9%)	9 (1.1%)	.802
Renal replacement therapy	21 (2.6%)	406 (3.5%)	.202	21 (2.6%)	19 (2.4%)	.873
Postoperative hospitalization (days)	7 (5-9)	7 (5-11)	<.001	7 (5-9)	7 (5-10)	.056
Post-discharge outcomes						
1-month mortality	9 (1.1%)	430 (3.7%)	<.001	9 (1.1%)	12 (1.5%)	.660
6-month mortality	20 (2.5%)	721 (6.3%)	<.001	20 (2.5%)	29 (3.6%)	.481
1-year mortality	25 (3.1%)	876 (7.6%)	<.001	25 (3.1%)	35 (4.4%)	.524
3-year mortality	57 (7.1%)	1458 (12.7%)	<.001	57 (7.1%)	72 (9.0%)	.365
5-year mortality	86 (10.7%)	1999 (17.4%)	<.001	86 (10.7%)	97 (12.1%)	.641

### Subgroup analysis of emergency surgery

Emergency surgery is an important part of a surgeon’s skillset to demonstrate competence and readiness for independent consultant practice. To assess the safety of independent resident-led emergency cardiac surgery, a subgroup analysis was performed. Autonomous resident-led emergency cases were propensity matched to consultant-led emergency cases, with preoperative characteristics (**Supplementary Table 1**), operative characteristics (**Supplementary Table 2**), and postoperative outcomes (**Supplementary Table 3**) presented.

**Figure 3:**
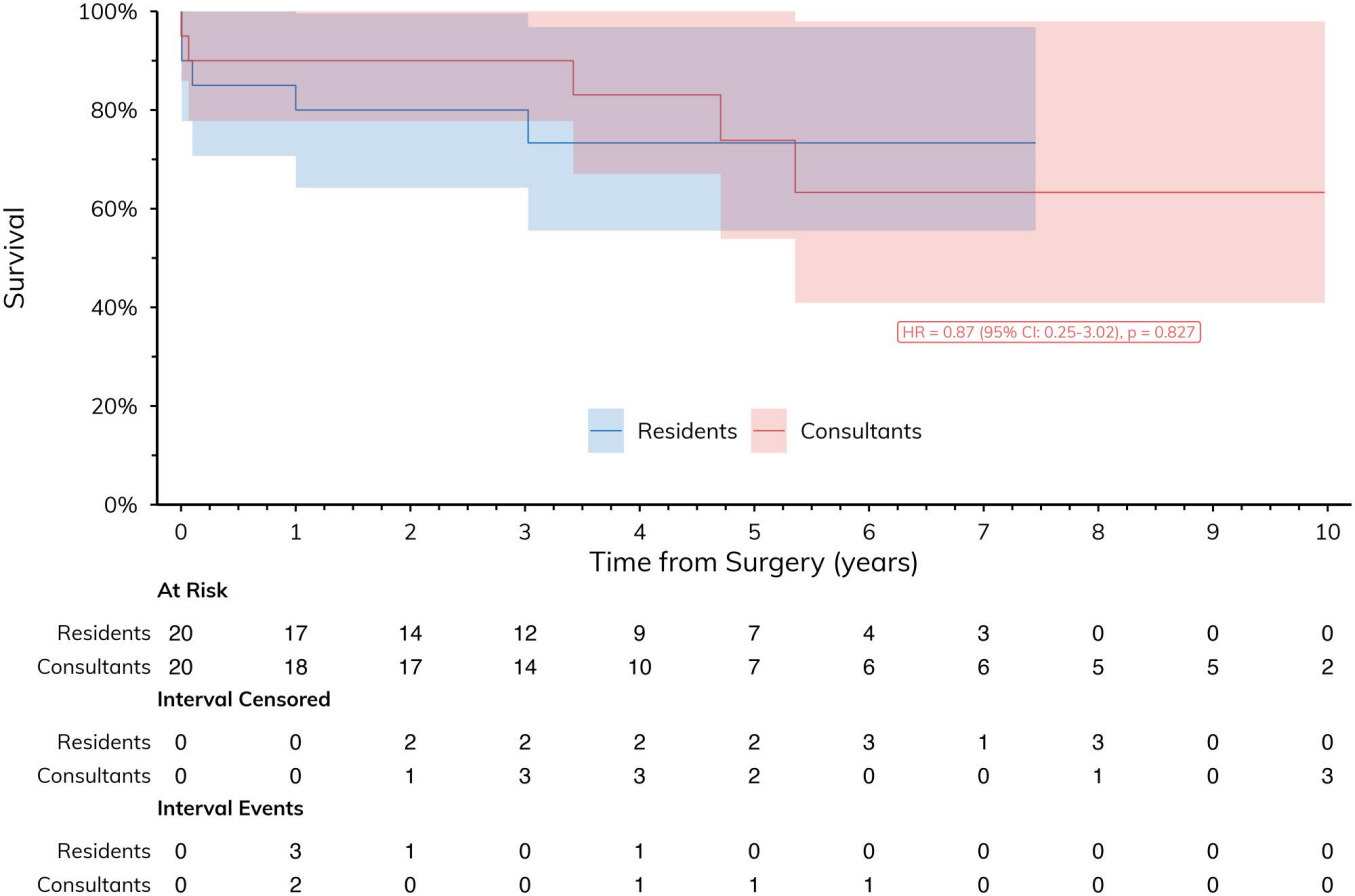
Long-Term Survival of the Emergency Cases Subgroup. Median follow-up duration was 44.1 months (IQR 30.8-68.7).

Groups R and C had similar operative characteristics. Rates of in-hospital complications were similar between groups, as was the postoperative length of hospital stay between groups. Long-term survival was comparable between groups (**Figure 3**). These findings suggest that, with appropriate selection and oversight, senior residents can safely perform emergency cardiac surgery with outcomes equivalent to those achieved by experienced consultants.

## DISCUSSION

This propensity-matched study demonstrates the safety and efficacy of autonomous cardiac surgery by senior residents. We demonstrate that these cases achieve comparable in-hospital and long-term outcomes to matched consultant-led procedures, with comparable mortality, complications, and survival. Similar complication rates are notable given their association with poor long-term prognosis.^18–21^

These results align with the recommendations of the EACTS Core Curriculum,^22^ that senior residents should perform routine cardiac procedures independently, achieving level 4-5 independence (indirect supervision to fully autonomous) by the completion of their training. It emphasises that they should be able to regularly perform a routine cardiac surgical procedure independently. Our study provides empirical validation of this training philosophy, demonstrating that senior residents performing these procedures autonomously achieve outcomes equivalent to consultant-led cases. This success may be attributed to excellent progressive training beforehand, enabling residents to ultimately reach this level of independence.

The inclusion of 803 resident-led cases across multiple surgeons underscores that these positive results reflect robust institutional training standards rather than exceptional individual performance, demonstrating that a structured progressive autonomy model produces safe independent surgeons. Propensity-score matching using EuroSCORE II covariates mitigates the selection bias that has confounded prior studies,^10^ and our analysis includes survival data up to 10 years, with many published studies lacking extended follow-up. Equivalent long-term survival is encouraging, affirming the safety of resident autonomy in appropriately selected cases.

Several notable findings warrant discussion. In the matched cohort, CPB and ACC times were similar between groups, suggesting that senior residents can operate efficiently under indirect supervision. These senior residents typically have completed at least 6 years of cardiac surgery training with over 150 cases as the primary operator under direct supervision. Case selection patterns offer further insights. Consultants performed more complex procedures, including combined CABG + valve (22.0% vs 14.6%) and multivalve operations (5.0% vs 1.7%), while residents handled more isolated CABG cases (56.5% vs 46.3%). Although matching generally resulted in the groups becoming more comparable, this was not reflected in the operative urgency. Residents also undertook significantly more in-house urgent cases (42.2% vs 35.7%), with consultants handling more elective (60.3% vs 55.3%) and emergency procedures (4.0% vs 2.5%). This distribution reflects appropriate case allocation, tailoring complexity to resident experience and aligning with graduated autonomy frameworks like the JCST supervision levels (progressing from direct to indirect supervision).^23^ Such strategies ensure patient safety while preparing senior residents for independent consultant practice.

An additional observation supporting the safety of this training model is that intraoperative escalation requiring consultants to scrub in and assist was uncommon. Of the cases intended to be undertaken autonomously by residents and commenced as such (*n* = 917), only a small proportion required a consultant to scrub partway through the procedure (*n* = 114, 12.4%). This finding suggests that case selection and supervisory thresholds were generally appropriate, with escalation occurring selectively when required rather than routinely. While the present study was not designed to analyse the specific indications or timing of intraoperative takeover, these events likely represent an important component of graduated surgical autonomy.

The subgroup analysis of emergency cases further validates these findings in high-stakes settings. Comparable in-hospital and post-discharge outcomes between groups support autonomous operating by the most senior residents, a challenging skill to develop. Our results align with equivalent outcomes with direct supervision^15^ and exceed the JCST expectations for level 3-4 emergency proficiency (able to act with indirect supervision to day 1 consultant level) by certification.

With 803 matched pairs, our study was adequately powered to detect clinically meaningful differences in primary outcomes. Confidence intervals for in-hospital mortality (OR 0.50, 95% CI 0.19-1.33) and long-term survival (HR 1.05, 95% CI 0.80-1.37) exclude clinically significant effect sizes, supporting equivalence between groups. For common complications such as return to theatre, CIs provided reasonable precision (OR 1.00, 95% CI 0.46-2.16). Rarer events like deep sternal wound infection showed wider CIs (OR 1.57, 95% CI 0.61-4.05), reflecting limited power for low-frequency outcomes, though point estimates consistently favoured neither group. Our sensitivity analysis using conditional logistic regression corroborated all primary findings, strengthening confidence in our conclusions. The emergency subgroup (20 pairs) had reduced precision with wider CIs. It was thus only powered to detect very large differences (absolute risk difference ∼22%, odds ratio ∼4.2).

In summary, this study demonstrates that truly autonomous cardiac surgery by senior residents is safe and effective, with equivalent outcomes to consultant-led cases, even in emergencies. These findings provide evidence-based support for graded independence in training, encouraging programs to leverage supervised opportunities, simulation, and competency metrics to prepare residents for independent practice while prioritizing patient safety.

### Limitations

This is a single-centre study from a high-volume unit with a long tradition of cardiac surgical training, limiting its generalizability to lower-volume centres and different training structures. Despite current guidance defining the term “first operator,” there is possibly variation in how this was defined from case to case. Furthermore, the differences in complexity between independent resident and consultant cases make it more challenging to draw reliable conclusions on whether resident first-operators are as efficient as consultants.

Unmeasured confounding remains possible as our matching model did not capture confounders such as poor mobility, pulmonary hypertension, or factors not included in EuroSCORE II, eg, frailty. The study period included major changes in the landscape of cardiac surgery, such as the switch to exclusively integrated training in the United Kingdom, affecting the level of experience a resident has at the start of their cardiac surgical residency. Although paired tests were not used, variance estimation accounted for matching; however, exploratory analyses lacked multiple comparison adjustments. Subgroups, especially the emergency cases with limited sample size, were underpowered for rarer outcomes, requiring cautious interpretation.

### Future directions

Multicentre studies, encompassing institutions with varied training structures, case volumes, and geographical locations, would significantly enhance the generalizability of these findings. Furthermore, longitudinal tracking of resident and surgeon performance throughout their training progression could pinpoint the optimal timing for residents to transition to more complex procedures and independent operating, such as cases included in this study. Such research would also delineate the learning curves for trained surgeons. Ultimately, this approach supports the development of robust, competency-based training milestones and assessment tools, ensuring both educational efficacy and patient safety, while also contributing to improved resident satisfaction through structured and supportive learning environments. Future work examining the predictors, timing, and outcomes of intraoperative escalation may provide further insight into how autonomy can be safely expanded within structured training environments. Finally, investigations into the long-term impact of autonomous resident surgery on career satisfaction and attrition rates within cardiac surgery could also provide valuable insights into sustainable training models.

## CONCLUSIONS

This comprehensive study demonstrates the safety and efficacy of autonomous cardiac surgical procedures performed by senior residents, demonstrating outcomes comparable to those achieved by consultants. These findings strongly advocate for judiciously implementing graduated autonomy in cardiac surgical training, emphasizing appropriate case selection and robust supervisory frameworks. Future work is necessary to develop more objective assessments to judge readiness for autonomous operating towards the end of cardiac surgical residency. In the right environment and with careful case selection, truly autonomous operating by senior residents is not only feasible; it is safe and essential to train tomorrow’s cardiac surgeons.

## Supplementary Material

ivag099_Supplementary_Data

## Data Availability

Data not publicly available for privacy reasons, but available on request to the corresponding author.

## Abbreviations

ACC: Aortic cross clamp
CABG: Coronary artery bypass grafting
CPB: Cardiopulmonary bypass
EuroSCORE: European System for Cardiac Operative Risk Evaluation
