# Supplementary material for: Comparing Early and Long-Term Outcomes of “Truly Autonomous” Senior Resident-Led with Consultant-Led Cardiac Surgery: A 10-Year Propensity-Matched Study
Source: Interdiscip Cardiovasc Thorac Surg. 2026 Apr 9;41(5):ivag099. doi: 10.1093/icvts/ivag099 (PMC13148963; doi:10.1093/icvts/ivag099)
Supplement: ivag099_Supplementary_Data [file ivag099_supplementary_data.pdf]

Supplementary Materials Legends

- Supplementary Table 1: Preoperative data (emergency cases)
- Supplementary Table 2: Operative data (emergency cases)
- Supplementary Table 3: Postoperative outcomes (emergency cases)
- Supplementary Figure 1: Matching plot.
- Appendix 1: Surgical consultants and residents throughout the study period.

Supplementary Tables

Supplementary Table 1. Preoperative characteristics of the study groups for emergency cases. *CrCl: creatinine clearance, CCS: Canadian Cardiovascular Society, LV: left ventricle, NYHA: New York Heart Association, BMI: body mass index.*

Variable	Unmatched Data				Matched Data			
	Residents N=20	Consultants N=834	p-value	SMD	Group R (Residents) N=20	Group C (Consultants) N=20	p-value	SMD
Age (years)	65 ± 10	64 ± 14	0.679	0.079	65 ± 10	63 ± 11	0.560	0.186
Sex: female	5 (25.0%)	266 (31.9%)	0.681	0.151	5 (25.0%)	3 (15.0%)	0.693	0.246
Chronic lung disease	3 (15.0%)	105 (12.6%)	>0.999	0.069	3 (15.0%)	5 (25.0%)	0.693	0.246
Extracardiac arteriopathy	3 (15.0%)	141 (16.9%)	>0.999	0.051	3 (15.0%)	6 (30.0%)	0.449	0.356
Previous cardiac surgery	1 (5.0%)	140 (16.8%)	0.831	0.383	1 (5.0%)	0 (0.0%)	>0.999	0.316
1	1 (5.0%)	92 (11.0%)			1 (5.0%)	0 (0.0%)		
2	0 (0.0%)	32 (3.8%)			0 (0.0%)	0 (0.0%)		
3	0 (0.0%)	3 (0.4%)			0 (0.0%)	0 (0.0%)		
4	0 (0.0%)	10 (1.2%)			0 (0.0%)	0 (0.0%)		
5	0 (0.0%)	3 (0.4%)			0 (0.0%)	0 (0.0%)		
6	0 (0.0%)	5 (0.0%)			0 (0.0%)	0 (0.0%)		
Critical preoperative state	3 (15.0%)	266 (31.9%)	0.173	0.403	3 (15.0%)	2 (10.0%)	>0.999	0.148
Preoperative CrCl (ml/min)	75 (58-99)	72 (51-98)	0.413	0.320	75 (58-99)	97 (66-118)	0.377	0.010
Renal impairment	8 (40.0%)	452 (54.2%)	0.483	0.396	8 (40.0%)	7 (35.0%)	0.988	0.061
CrCl 50-85ml/min	6 (30.0%)	259 (31.1%)			6 (30.0%)	5 (25.0%)		
CrCl <50ml/min	1 (5.0%)	130 (15.6%)			1 (5.0%)	1 (5.0%)		
Dialysis	1 (5.0%)	63 (7.6%)			1 (5.0%)	1 (5.0%)		
Diabetes mellitus	6 (30.0%)	184 (22.1%)	0.402	0.256	6 (30.0%)	4 (20.0%)	0.506	0.331
Lifestyle-controlled	0 (0.0%)	32 (3.8%)			0 (0.0%)	1 (5.0%)		
Oral medication	3 (15.0%)	94 (11.3%)			3 (15.0%)	2 (10.0%)		
Insulin-dependent	3 (15.0%)	58 (7.0%)			3 (15.0%)	1 (5.0%)		
CCS class			0.246	0.394			0.891	0.178
1	8 (40.0%)	479 (57.4%)			8 (40.0%)	7 (35.0%)		
2	2 (10.0%)	74 (8.9%)			2 (10.0%)	1 (5.0%)		
3	2 (10.0%)	101 (12.1%)			2 (10.0%)	2 (10.0%)		
4	8 (40.0%)	180 (21.6%)			8 (40.0%)	10 (50.0%)		
LV dysfunction	8 (40.0%)	355 (42.6%)	0.782	0.171	8 (40.0%)	8 (40.0%)	0.659	0.183
Mild dysfunction	6 (30.0%)	221 (26.5%)			6 (30.0%)	4 (20.0%)		
Moderate dysfunction	2 (10.0%)	95 (11.4%)			2 (10.0%)	3 (15.0%)		
Severe dysfunction	0 (0.0%)	39 (4.7%)			0 (0.0%)	1 (5.0%)		
Previous myocardial infarction	8 (40.0%)	269 (32.3%)	0.624	0.160	8 (40.0%)	10 (50.0%)	0.751	0.197
Myocardial infarction within 90d	8 (40.0%)	238 (28.5%)	0.385	0.240	8 (40.0%)	9 (45.0%)	>0.999	0.099
NYHA class			0.561	0.251			0.276	0.328
1	10 (50.0%)	293 (35.1%)			10 (50.0%)	5 (25.0%)		
2	4 (20.0%)	197 (23.6%)			4 (20.0%)	6 (30.0%)		
3	3 (15.0%)	201 (24.1%)			3 (15.0%)	7 (35.0%)		
4	3 (15.0%)	143 (17.1%)			3 (15.0%)	2 (10.0%)		
Body mass index (kg/m^2)	28.67 ± 7.87	27.89 ± 9.07	0.670	0.091	28.67 ± 7.87	27.21 ± 5.10	0.492	0.220
Smoking history	8 (40.0%)	400 (48.0%)	0.780	0.144	8 (40.0%)	12 (60.0%)	0.416	0.411
Ex-smoker	6 (30.0%)	300 (36.0%)			6 (30.0%)	8 (40.0%)		
Current smoker	2 (10.0%)	100 (12.0%)			2 (10.0%)	4 (20.0%)		
Neurological dysfunction	2 (10.0%)	86 (10.3%)	>0.999	0.010	2 (10.0%)	3 (15.0%)	>0.999	0.148
Hypertension	10 (50.0%)	446 (53.5%)	0.935	0.069	10 (50.0%)	17 (85.0%)	0.043	0.785
Unstable angina	8 (40.0%)	149 (17.9%)	0.034	0.478	8 (40.0%)	9 (45.0%)	0.888	0.145

Supplementary Table 2. Operative characteristics of the study groups for emergency cases. *CABG: coronary artery bypass graft*

Variable	Unmatched Data			Matched Data		
	Residents N=20	Consultants N=834	p-value	Group R (Residents) N=20	Group C (Consultants) N=20	p-value
Logistic EuroSCORE	24.95 (6.92-40.39)	25.36 (11.94-44.66)	0.238	24.95 (6.92-40.39)	21.20 (10.38-30.56)	0.745
EuroSCORE II	4.61 (2.03-12.49)	8.71 (4.02-20.01)	0.089	4.61 (2.03-12.49)	6.45 (3.08-11.62)	0.776
Procedure type			0.454			0.856
Aortic	7 (35.0%)	359 (43.0%)		7 (35.0%)	7 (35.0%)	
CABG	10 (50.0%)	220 (26.4%)		10 (50.0%)	10 (50.0%)	
CABG + valve	2 (10%)	84 (10.1%)		2 (10.0%)	1 (5.0%)	
Valve	1 (5%)	142 (17%)		1 (5.0%)	2 (10.0%)	
Multivalve	0 (0.0%)	29 (3.5%)		0 (0.0%)	0 (0.0%)	
Median number of distal anastomoses	2 (2-3)	3 (2-3)	0.413	2 (2-3)	3 (2-3)	0.246
Cardiopulmonary bypass time (minutes)	96 (65-127)	150 (96-221)	0.006	96 (65-127)	146 (94-187)	0.096
Aortic cross-clamp time (minutes)	58 (37-66)	81 (53-118)	0.003	58 (37-66)	80 (52-100)	0.056

Supplementary Table 3. Postoperative outcomes of the study groups for emergency cases.

Variable	Unmatched Data			Matched Data		
	Residents N=20	Consultants N=834	p-value	Group R (Residents) N=20	Group C (Consultants) N=20	p-value
**In-Hospital Outcomes**						
Hospital mortality	2 (10.0%)	132 (15.8%)	0.691	2 (10.0%)	2 (10.0%)	>0.999
Return to theatre	2 (10.0%)	84 (10.1%)	0.657	2 (10.0%)	0 (0.0%)	0.349
Deep sternal wound infection	0 (0.0%)	5 (0.6%)	>0.999	0 (0.0%)	0 (0.0%)	>0.999
Stroke	3 (15.0%)	62 (7.4%)	0.404	3 (15.0%)	0 (0.0%)	0.230
Renal replacement therapy	6 (30.0%)	119 (14.3%)	0.100	6 (30.0%)	1 (5.0%)	0.096
Postoperative hospitalisation (days)	7 (6-12)	11 (6-17)	0.068	7 (6-12)	8 (6-10)	0.649
**Post-Discharge Outcomes**						
One-month mortality	2 (10.0%)	157 (18.8%)	0.477	2 (10.0%)	2 (10.0%)	>0.999
Six-month mortality	3 (15.0%)	208 (24.9%)	0.450	3 (15.0%)	2 (10.0%)	>0.999
One-year mortality	3 (15.0%)	220 (26.4%)	0.375	3 (15.0%)	2 (10.0%)	>0.999
Three-year mortality	4 (20.0%)	272 (32.6%)	0.342	4 (20.0%)	2 (10.0%)	0.658
Five-year mortality	5 (25.0%)	307 (36.8%)	0.396	5 (25.0%)	4 (20.0%)	>0.999

Supplementary Figures

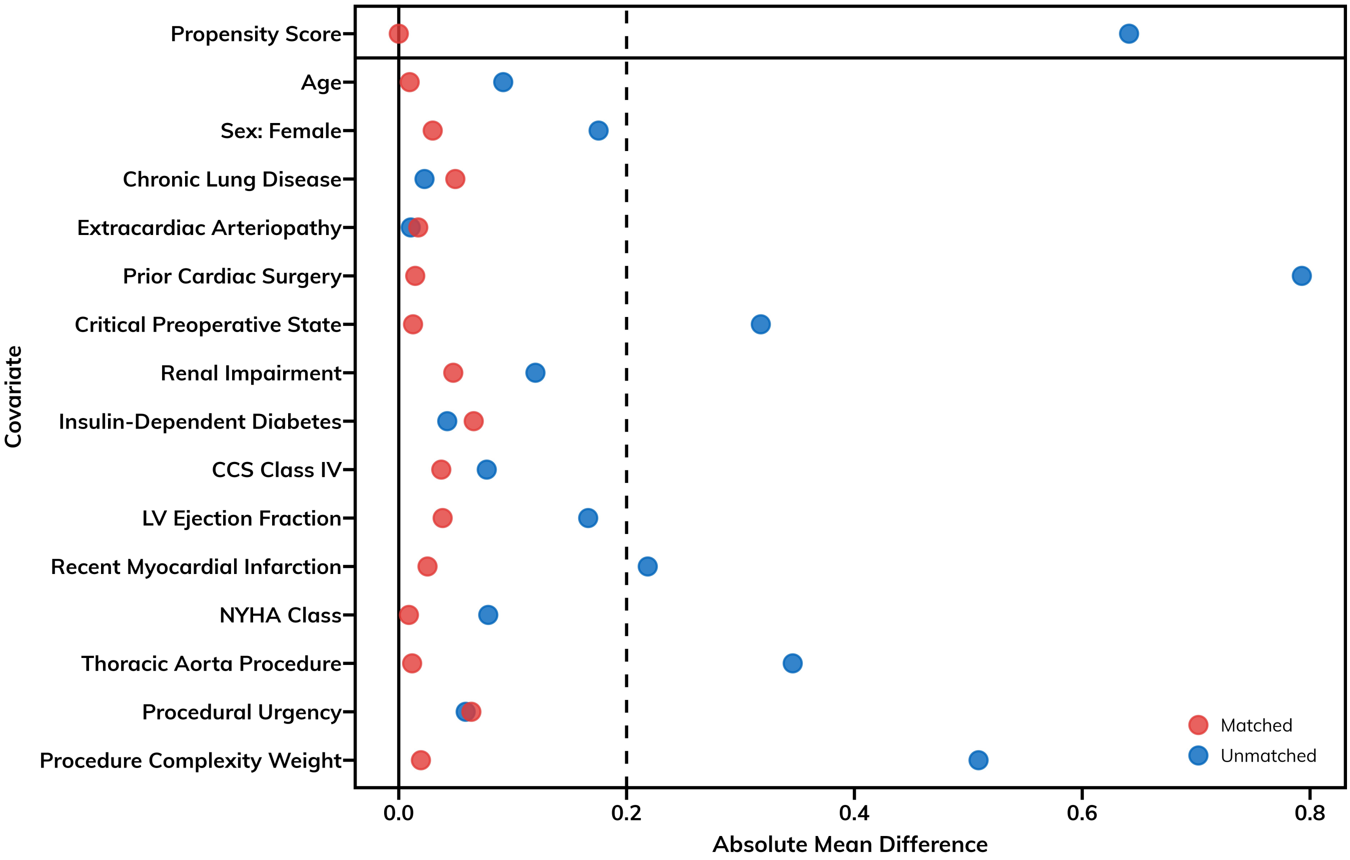

Supplementary Figure 1: Love plot demonstrating that groups were well-matched across all covariates following propensity-matching.

Appendix 1

This work would not have been possible without all of the consultant cardiothoracic surgeons across the study period. We would like to thank them for their contributions to training the next generation of cardiac surgeons. Thanks are also due to all of the surgical residents and all staff (past and present) at Royal Papworth Hospital for their committment to patient care.

Consultants

Yasir Abu-Omar, Ayyaz Ali, Jason Ali, Rizwan Attia, Marius Berman, Pedro Catarino, Ravi De Silva, Vamsidhar Dronavilli, John Dunning, Shakil Farid, Swetha Iyer, David Jenkins, Pradeep Kaul, Stephen Large, Prashant Mohite, Narain Moorjani, Sam Nashef, Choo Ng, Nishith Patel, Umar Rafiq, Hassiba Smail, Catherine Sudarshan, Fouad John Taghavi, Steven Tsui, Francis Wells.

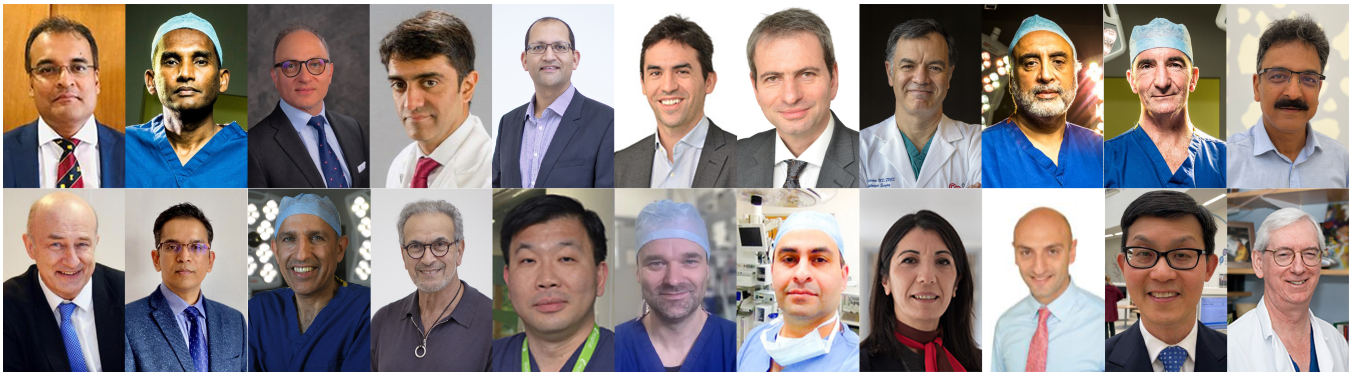

Residents

Ahmed Al-Adhami, Ruhina Alam, Jason Ali, Lubna Bakr, Paolo Bosco, Pedro Catarino, Stephen Chiu, Vamsidhar Dronavilli, Walid Elmahdy, Fabio Falconieri, Shakil Farid, Antonella Ferrara, Swetha Iyer, Jay Karunanantham, Simon Messer, Ashvin Nair, Prakash Nanjaiah, Manoraj Navaratnarajah, Alia Noorani, Mohamed Osman, Aravinda Page, Rushmi Purmessur, Umar Rafiq, Taufiek Rajab, Rakesh Seetharaman, Thomas Senage, Ahmed Shafi, Daniel Sitaranjan, Fouad John Taghavi, Ismail Vokshi, Ivan Yim.
